# Identifying Adolescents at Highest Risk of ART Non-adherence, Using the World Health Organization-Endorsed HEADSS and HEADSS+ Checklists

**DOI:** 10.1007/s10461-023-04137-6

**Published:** 2023-08-17

**Authors:** Lucie D. Cluver, Yulia Shenderovich, Marko Seslija, Siyanai Zhou, Elona Toska, Alice Armstrong, Laurie A. Gulaid, Wole Ameyan, Matteo Cassolato, Caroline C. Kuo, Christina Laurenzi, Lorraine Sherr

**Affiliations:** 1 Centre for Evidence-Based Intervention, Department of Social Policy & Intervention, University of Oxford, Barnett House, 32 Wellington Square, Oxford, OX1 2ER UK; 2 Wolfson Centre for Young People’s Mental Health, Cardiff University, Cardiff, UK; 3 Centre for the Development and Evaluation of Complex Interventions for Public Health Improvement (DECIPHer), School of Social Sciences, Cardiff University, Cardiff, UK; 4 Centre for Social Science Research, University of Cape Town, Cape Town, South Africa; 5 Division of Social and Behavioural Sciences, School of Public Health, Faculty of Health Sciences, University of Cape Town, Cape Town, South Africa; 6 UNICEF Eastern and Southern Africa Regional Office, Nairobi, Kenya; 7 Global HIV, Hepatitis and Sexually Transmitted Infections Programmes, World Health Organization, Geneva, Switzerland; 8 Frontline AIDS, Brighton, UK; 9 Department of Health Studies, American University, Washington, DC USA; 10 Department of Global Health, Institute for Life Course Health Research, Stellenbosch University, Stellenbosch, South Africa; 11 Health Psychology Unit, Institute of Global Health, University College London, London, UK; 12 Department of Psychiatry and Mental Health, University of Cape Town, Cape Town, South Africa

**Keywords:** Adolescents, HIV, AIDS, Mental health, Health personnel, Treatment

## Abstract

Brief tools are necessary to identify adolescents at greatest risk for ART non-adherence. From the WHO’s HEADSS/HEADSS+ adolescent wellbeing checklists, we identify constructs strongly associated with non-adherence (validated with viral load). We conducted interviews and collected clinical records from a 3-year cohort of 1046 adolescents living with HIV from 52 South African government facilities. We used least absolute shrinkage and selection operator variable selection approach with a generalized linear mixed model. HEADSS constructs most predictive were: violence exposure (aOR 1.97, CI 1.61; 2.42, p < 0.001), depression (aOR 1.71, CI 1.42; 2.07, p < 0.001) and being sexually active (aOR 1.80, CI 1.41; 2.28, p < 0.001). Risk of non-adherence rose from 20.4% with none, to 55.6% with all three. HEADSS+ constructs were: medication side effects (aOR 2.27, CI 1.82; 2.81, p < 0.001), low social support (aOR 1.97, CI 1.60; 2.43, p < 0.001) and non-disclosure to parents (aOR 2.53, CI 1.91; 3.53, p < 0.001). Risk of non-adherence rose from 21.6% with none, to 71.8% with all three. Screening within established checklists can improve identification of adolescents needing increased support. Adolescent HIV services need to include side-effect management, violence prevention, mental health and sexual and reproductive health.

**Supplementary Information:**

The online version contains supplementary material available at 10.1007/s10461-023-04137-6.

## Introduction

Adolescents (aged 10–19 years) living with HIV are at substantially elevated risk of antiretroviral (ART) non-adherence [1]. Of the 1.7 million adolescents living with HIV globally, 91% live in Sub-Saharan Africa [2]. Many of these adolescents access HIV services from overburdened health systems, receiving care via decentralised primary health clinics with few or no specialist providers, and from health workers with limited time. Shifts towards multi-month dispensing of ART may also reduce regularity of adolescent interactions with the HIV healthcare system [3].

In these contexts, it is essential to develop innovative ways to identify adolescents at greatest risk of non-adherence and ART discontinuation. However, self-reported adherence is often unreliable, and other adherence measurement approaches have low effectiveness across age groups [4], such as pill-counting [5]. HIV viral load testing rates remain low across the region [6], and technologies such as electronic monitoring through digital pill caps are not yet feasible at scale in low-resource settings.

To identify and support adolescents who are at higher risk of non-adherence, we need simple, adolescent-friendly, and acceptable screening methods that a range of providers can use. These methods should be time-efficient and user-friendly to improve feasibility and scalability. In examining checklists for non-adherence, Lowenthal et al. [7] identified family support, self-efficacy, future aspirations [7], and psychological reactance to reminders [8] as key factors in Botswana. Valuable checklists such as the Pediatric Symptom Checklist have been found to be associated with virologic failure in the U.S. and sub-Saharan Africa [9], but a need remains for very brief routine screening in high-burden, under-resourced settings.

One approach is to identify critical components of established tools that are already widely used in clinical care. The Home, Education/employment, peer group Activities, Drugs, Sexuality, and Suicide/depression (HEADSS) checklist was developed in the 1970s and refined in the early 2000s [10, 11]. The World Health Organization recommends its use as a structured assessment of general adolescent psychosocial risk and wellbeing [12]. HEADSS has been used extensively in Sub-Saharan Africa with paediatric hospital populations [13, 14] and with adolescents living with HIV [15] (Fig. 3). In 2017, Frontline AIDS adapted HEADSS to include assessments that were specific to adolescents living with HIV, creating the HEADSS+ checklist. These linked checklists are non-commercial, freely accessible, and translated into multiple languages. There is variation in whether HEADSS and HEADSS+ are used together or as individual checklists in clinical and community settings. Rather than standardised items, they provide a series of constructs (for example around mental health, peer relationships, and sexuality), and support adaptability to local contexts—for instance, different questions may be used to operationalise depression symptoms across settings and healthcare providers [16].

In this study, we sought to identify the briefest possible sets of factors associated with adolescent ART non-adherence from the HEADSS and HEADSS+ checklists. For healthcare facilities and community organisations that already use the HEADSS or HEADSS+ checklists, these could allow identification of adolescents most at risk of non-adherence.

## Methods

### Sampling and Approach

The Mzantsi Wakho study took place in South Africa’s Eastern Cape, an area with fragile health systems, high HIV and TB, and poor infrastructure [17–19]. In a health district including peri-urban and rural settlements, we identified every government facility providing ART to paediatric/adolescent populations (n = 52). Across facilities (hospitals, primary clinics, community health centres), paper and electronic patient files were reviewed to identify all adolescents (10–19 years) who had ever initiated ART—whether currently in healthcare or not.

We used community-tracing to 180 settlements, interviewed adolescents at their preferred location, and extracted viral loads from their clinic files. At two subsequent follow-up periods (Wave 2, 18 months and Wave 3, 36 months), all adolescents who had consented to be re-approached were asked for consent for follow-up. At baseline (2014–2015), the sample included 1046 adolescents living with HIV. At Wave 2 (2016–2017), retention was 94% (n = 979), and at Wave 3 (2018–2019) it was 96% (n = 933). 3.4% of adolescents died over the 36 months. To prevent stigma, we also interviewed neighbour adolescents (n = 456, data omitted from these analyses), and presented the study locally as a general adolescent wellbeing survey. Reflecting high mobility, 18% of participants had moved households between study waves, and by follow-up participants lived in six provinces: Eastern Cape, Gauteng, KwaZulu-Natal, Free State, Western Cape, and North-West.

Ethics approvals were obtained from the University of Cape Town (CSSR 2013/4), Oxford University (CUREC2/12-21), Provincial Departments of Health and Education, National Health Laboratory Service (NHLS) Academic Affairs and Research Management System (2019/08/07) and healthcare facilities. All adolescents and their primary caregivers gave written informed consent at each time point in their preferred language (Xhosa or English), read aloud in cases of low literacy. Trained local researchers supported participants to complete tablet-based questionnaires lasting 60–90 min, in the adolescent’s preferred language. Questionnaire wording and content were co-designed with an adolescent advisory group [20]; the South African National Departments of Health, Social Development, Basic Education and National AIDS Council; UNICEF; PEPFAR-USAID, and local NGOs. Pre-piloting was conducted locally with 25 adolescents.

For their participation, adolescents received a snack, a certificate of participation, and a small gift pack including soap and pencils—recommended by our adolescent advisory group and provided regardless of interview completion. Confidentiality was maintained except in cases of risk of harm. For rape, abuse, suicidality, or untreated severe illness (e.g. symptomatic TB), researchers made immediate health and social service referrals with follow-up support (n = 246 referrals over 3 years for 157 adolescents).

### Identifying HEADSS and HEADSS+ Constructs

**Fig. 1:**
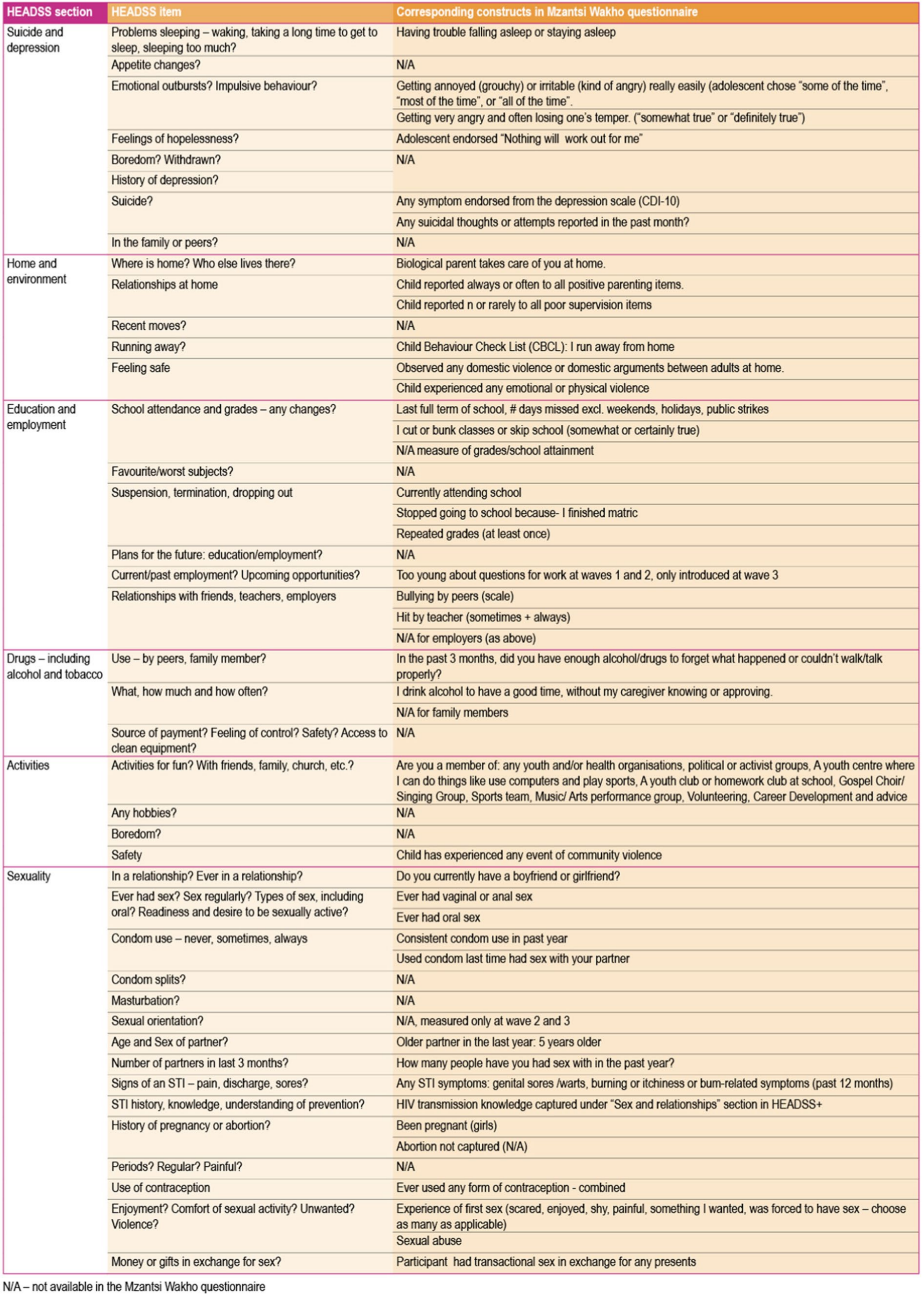
HEADSS constructs mapped onto the Mzantsi Wakho questionnaire

**Fig. 2:**
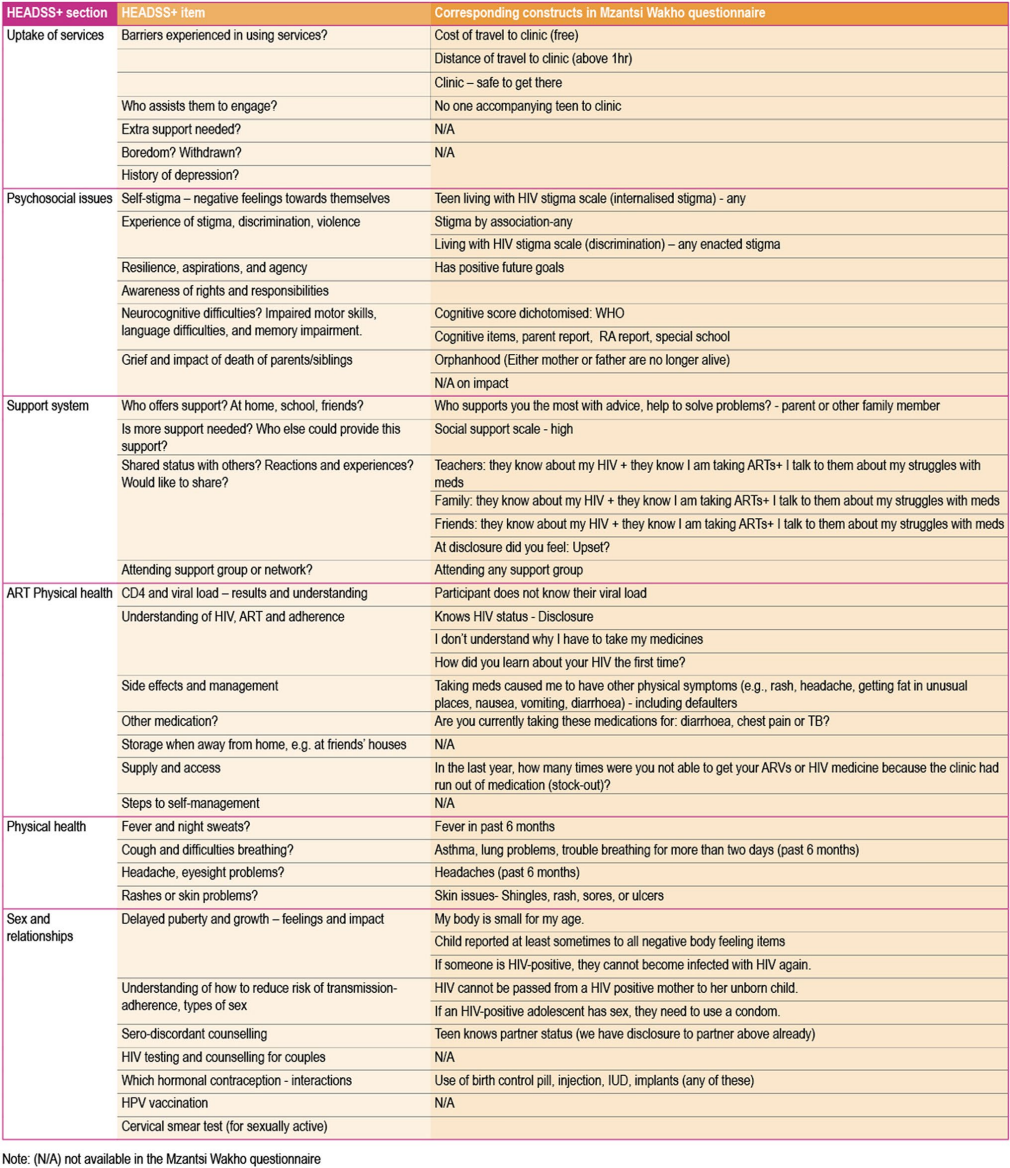
HEADSS+ constructs mapped onto the Mzantsi Wakho questionnaire

We mapped study variables alongside the HEADSS and HEADSS+ constructs [21], finding that almost all constructs were represented (Figs. 1, 2). All constructs were coded as binary for comparability across constructs. We also checked that variation was present for each included variable (> 5% of participants per category) and included only variables available at all three timepoints.

Prior to analyses, following recommendations in the variable selection literature [22], we examined all potential constructs to see if existing evidence suggested plausible associations with adherence [23, 24]. All constructs were plausibly correlated with adherence and therefore we focused on statistical methods to support variable selection.

### Study Measures

All variables were defined in the same way across three timepoints. *ART adherence* was measured using adapted items from the Patient Medication Adherence Questionnaire and measures developed in Botswana [7]. ART adherence was defined as past 7 days adherence > 95%. Viral load measures were obtained from data abstracted from patient clinic records, and routine biomarker data from South Africa’s NHLS following the linkage of participant’s sociodemographic data to the NHLS data warehouse.

### Possible Identifiers of Non-adherence

We assessed a total of 69 constructs aligned with HEADSS (33) and HEADSS+ (36), with all constructs described in Fig. 1. Full questionnaires are available *here*. The HEADSS sections include: home and environment; exposure to violence; education and employment; suicide and depression; sexuality; substance use; activities. HEADSS+ covers: physical health; ART experience; support system; psychosocial issues; uptake of services; sex and relationships.

### Statistical Analyses

First, we validated self-reported adherence against an undetectable viral load (< 50 copies/ml), and the viral load measurement closest to the interview date (< 12 months before or after, allocating to the closest interview wave) for all the three timepoints. Second, we identified the most predictive set of three constructs in each section. Stepwise variable selection methods can lead to overfitting, such that *R*^2^ and regression coefficient become inflated, while standard errors and *p* values become too low [25–27]. To combat this, we used the least absolute shrinkage and selection operator (Lasso) approach [28] for variable selection and robust regression [29–31]—see Supplementary Materials for more information.

In order to take into account person-specific characteristics and the clustered nature of the repeated measures data in this study, we fit a generalized linear mixed model with L1-penalty term that enforces variable selection and shrinkage simultaneously [30]. To enable derivation of a brief list of key constructs, feasible for use in practice, we selected the three top variables from each checklist. Rather than using information criteria, such as AIC and BICs, or cross-validation for λ selection, we tuned the λ parameter so that the Lasso algorithm selected the three factors most strongly associated with ART non-adherence. Since some healthcare settings use only HEADSS or HEADSS+, variable selection analyses were run separately for each checklist. We also conducted a sensitivity analysis regarding the variable selection: we treated all observations as independent of each other (i.e. not accounting for clustered nature of data) and conducted a standard Lasso as well as elastic net variable selection procedures.

With the selected sets of variables, we ran logistic random-intercept regressions to illustrate the average relationships of these variables with adherence across the three timepoints, including controls of participant sex and age. We use mixed-effects (random intercept) logistic regression to provide estimates of the relationships between the selected sets of three variables and non-adherence while modelling the repeated measures structure of the data (from the same subjects at three timepoints). Finally, we examine average adjusted predicted probabilities of ART non-adherence, based on the levels of the selected factors. We have made all R code available open-source online: https://github.com/marses/HEADSS.

## Results

**Fig. 3:**
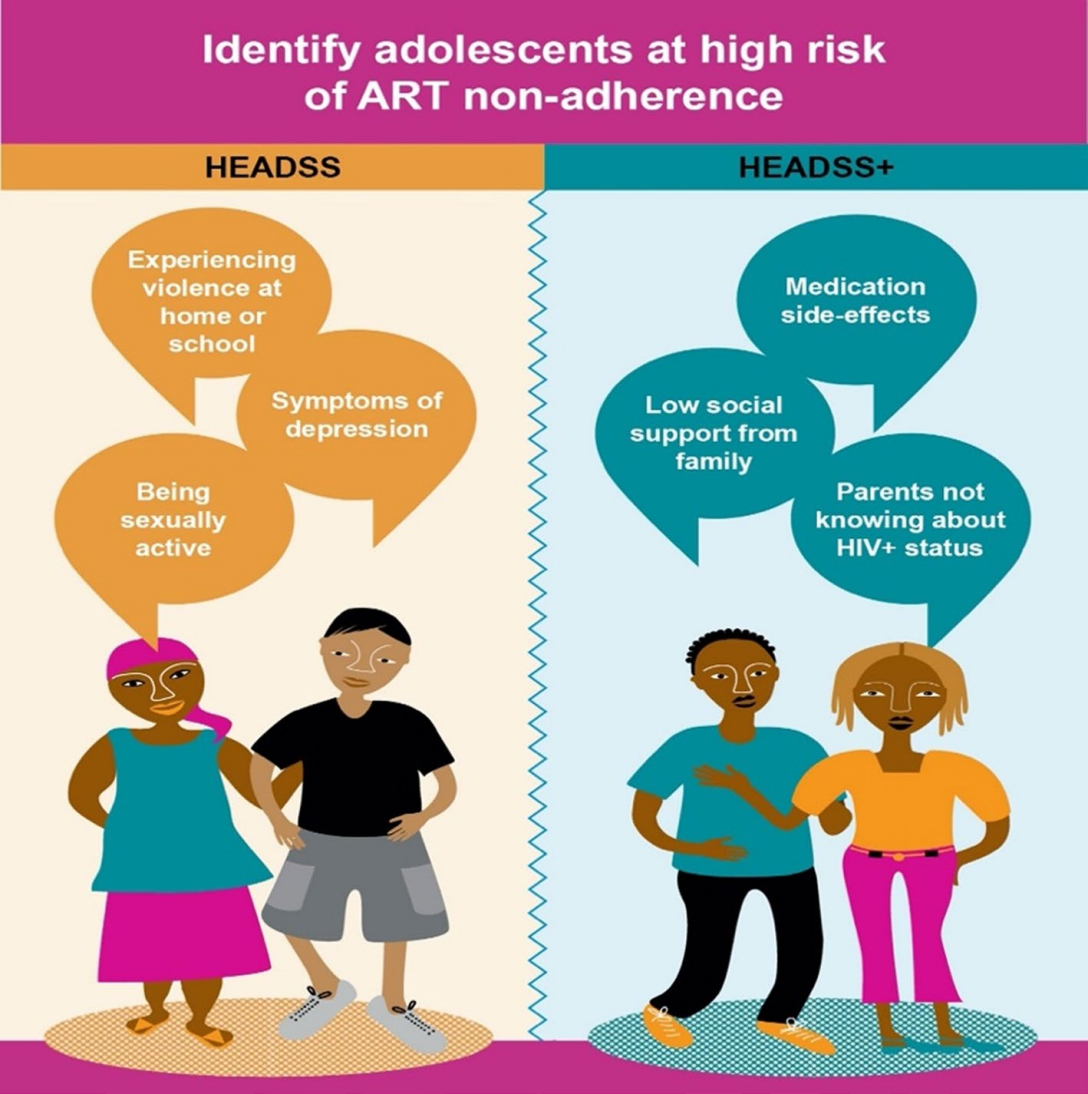
Items from the HEADSS and HEADSS+ checklist that can support identification of adolescents at highest risk of ART non-adherence

See Fig. 3 for a poster or memory aid for healthcare staff, following the HEADSS+ graphic style.

### Viral Load (VL) and Adherence

Past-week self-reported adherence was associated with undetectable viral load (< 50 copies/ml) (aOR 1.51, 95% CI 1.11; 2.05, p = 0.008), controlling for age, sex, rural/urban, double orphanhood, informal housing and mode of infection (see Table 1 Supp).

### HEADSS and HEADSS+ Constructs are Most Associated with Non-adherence

**Fig. 4:**
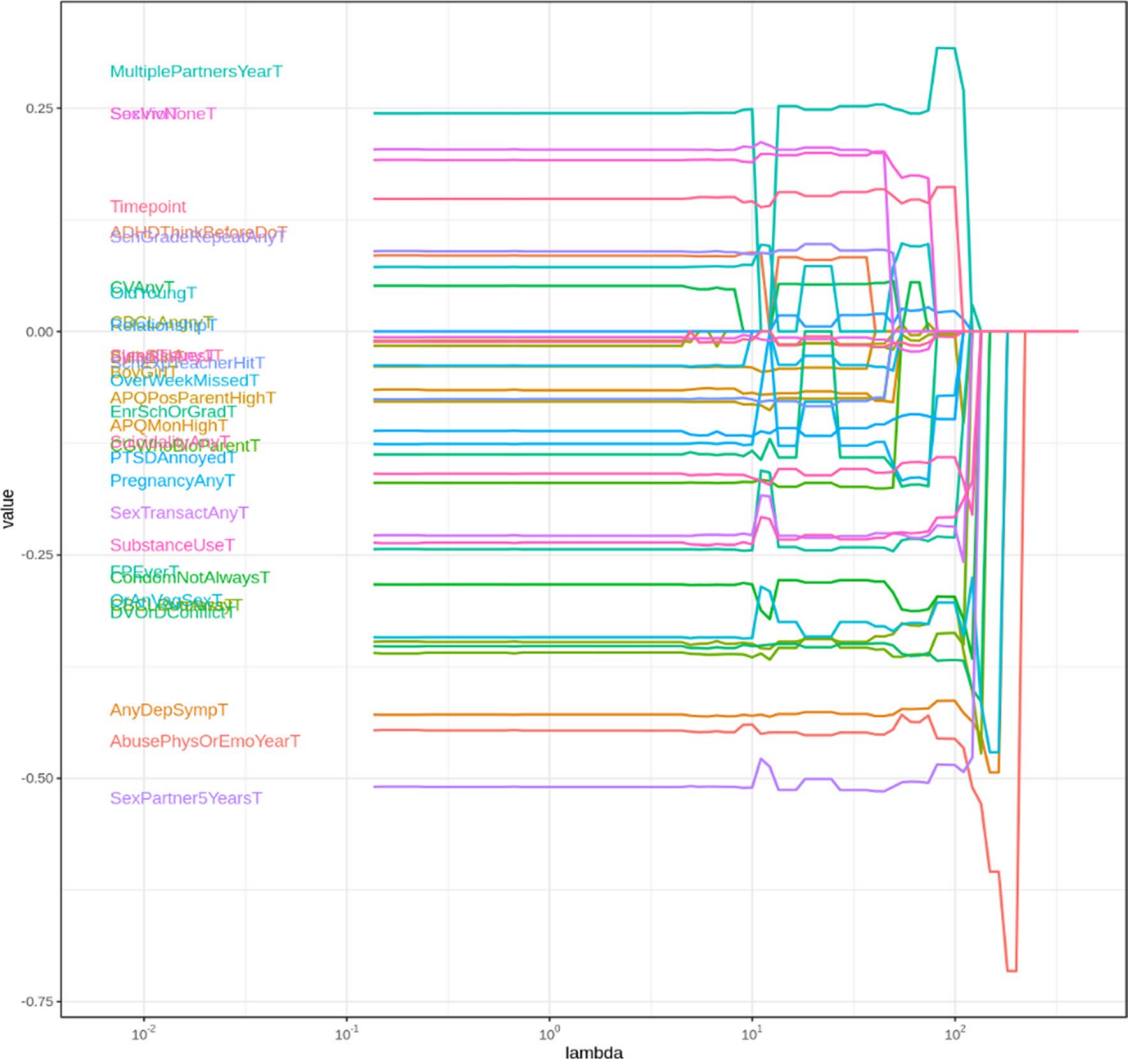
Coefficient build-ups for HEADSS dataset. The horizontal axis shows increasing (from left to right) values of the penalty coefficient λ, and the vertical axis shows the value of the regression coefficient for the given variable at the different levels of lambda. This figure and Fig. 5 illustrate how Lasso has selected variables as a function of the penalty parameter λ within the HEADSS and HEADSS sub-sets, respectively. Each curve on the plot corresponds to a single variable. For sufficiently large penalty λ, the only model selected is a model with only the intercept as all coefficients decrease to zero. As λ decreases, more variables are included in the model, which leads to all variables being included when λ becomes sufficiently small. The graphs show the path of model coefficients against λ. The first three paths that emerge on right are selected as three most important

**Fig. 5:**
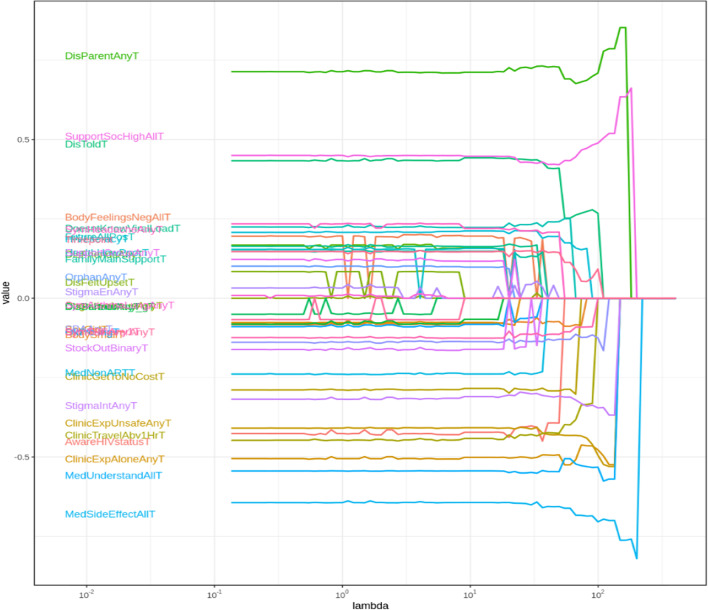
Coefficient build-ups for HEADSS+ dataset. The horizontal axis shows increasing (from left to right) values of the penalty coefficient λ, and the vertical axis shows the value of the regression coefficient for the given variable at the different levels of lambda. Figure 4 and this figure illustrate how Lasso has selected variables as a function of the penalty parameter λ within the HEADSS and HEADSS sub-sets, respectively. Each curve on the plot corresponds to a single variable. For sufficiently large penalty λ, the only model selected is a model with only the intercept as all coefficients decrease to zero. As λ decreases, more variables are included in the model, which leads to all variables being included when λ becomes sufficiently small. The graphs show the path of model coefficients against λ. The first three paths that emerge on right are selected as three most important

**Fig. 6:**
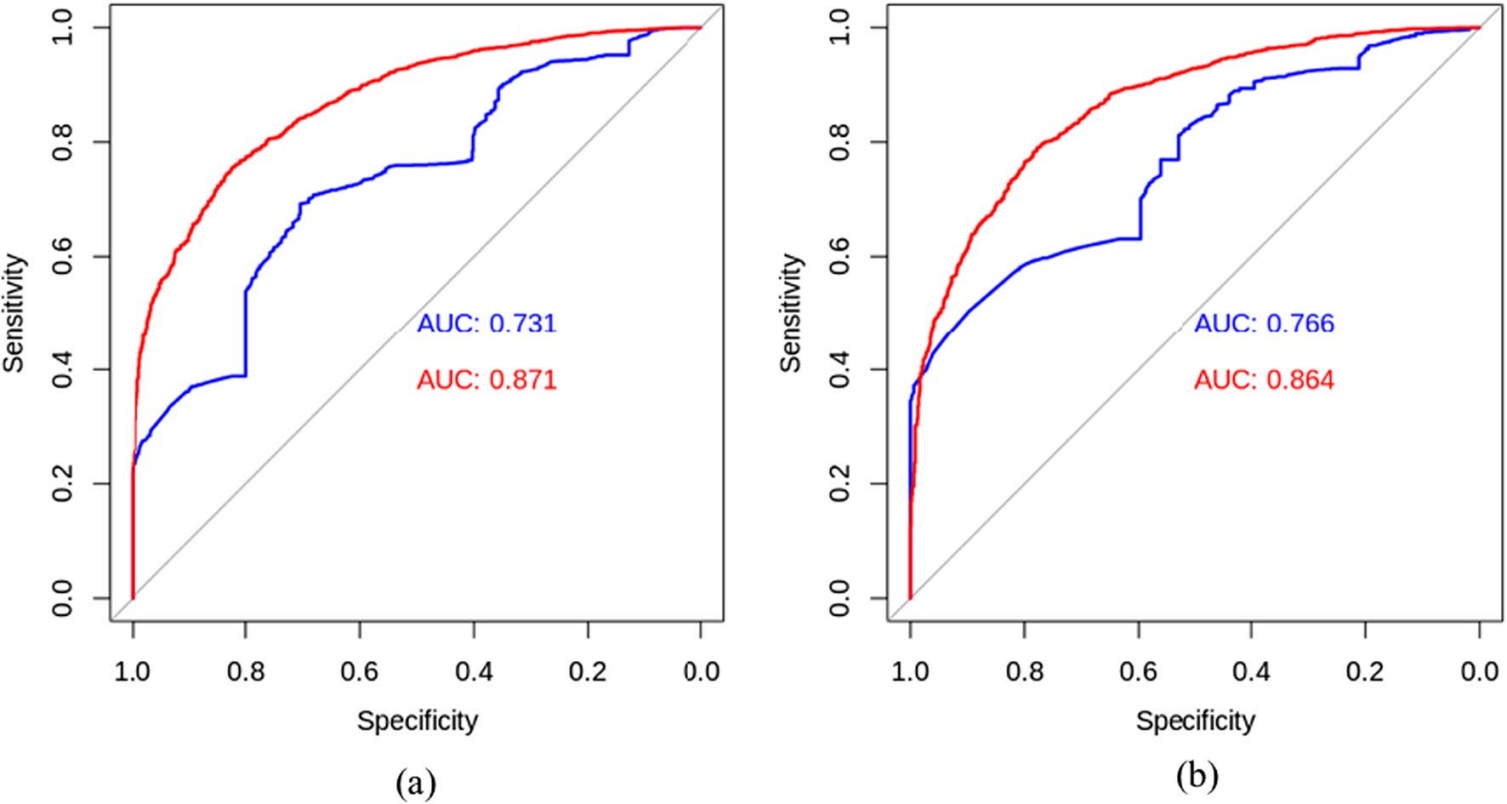
ROC curve of the full model (red) and of the 3-variable model selected by Lasso (blue). The panel on the left **a** shows results for HEADSS dataset and the panel on the right **b** shows results for HEADSS+ dataset. This figure illustrates the receiver operating characteristic (ROC) curves for both datasets. The area under the curve (AUC) for the model with three top HEADSS variables was 0.731 and with HEADSS+ it was 0.766. For comparison, the full HEADSS model (including all variables in Fig. 1) AUC was 0.871 and for the HEADSS+ model AUC was 0.864 (Color figure online)

Lasso selection identified the following set of highest-performing three variables from each checklist that were associated with adherence (reported in reverse for association with non-adherence). From HEADSS, these were: exposure to recent violence, depression symptoms, and being sexually active. From HEADSS+, these were: reporting ART side effects, low social support, and parents/caregivers unaware of adolescent’s HIV status or ART usage. The selected λ value for HEADSS was 155 and for HEADSS+ 170 (Figs. 4, 5, 6).

### Multivariable Associations Between Selected Factors and Non-adherence

Using the non-penalised regression results from Table 1, the three identified constructs for HEADSS: emotional or physical violence exposure (aOR 1.97, 95%CI 1.61; 2.42, p < 0.001), experiencing any depression symptoms (aOR 1.71, 95%CI 1.42; 2.07, p < 0.001) and being sexually active (aOR 1.80, 95%CI 1.41; 2.28, p < 0.001) were significantly associated with higher likelihood of non-adherence. For HEADSS+, experiencing medication side effects (aOR 2.27, 95%CI 1.82; 2.81, p < 0.001), low social support (aOR 1.97, 95%CI 1.60; 2.43, p < 0.001) and parent not knowing adolescent’s HIV status (aOR 2.53, 95%CI 1.91; 3.53, p < 0.001) was associated with a higher likelihood of non-adherence.

**Table 1:** Regressions demonstrating associations of the selected variables and non-adherence

Explanatory variable	Non-penalised regression	
	Odds ratio (95% CI)	p-value
A. HEADSS aligned characteristics (N = 933)		
Emotional or physical violence exposure	1.97 (1.61; 2.42)	< 0.001
Depression symptoms	1.71 (1.42; 2.07)	< 0.001
Sexually active	1.80 (1.41; 2.28)	< 0.001
Intercept	0.60 (0.30; 1.19)	0.144
B. HEADSS+ aligned characteristics (N = 916)		
ARTs/medication side effects	2.27 (1.82; 2.81)	< 0.001
Low social support	1.97 (1.60; 2.43)	< 0.001
Parent does not know about adolescent’s HIV status	2.53 (1.91; 3.53)	< 0.001
Intercept	0.43 (0.24; 0.77)	0.005

Penalised models include the full set of variables. Control variables in non-penalised regression models are age and gender/sex. P-values for non-penalised regression and predicted probabilities may be particularly small due to the previous variable selection

**Fig. 7:**
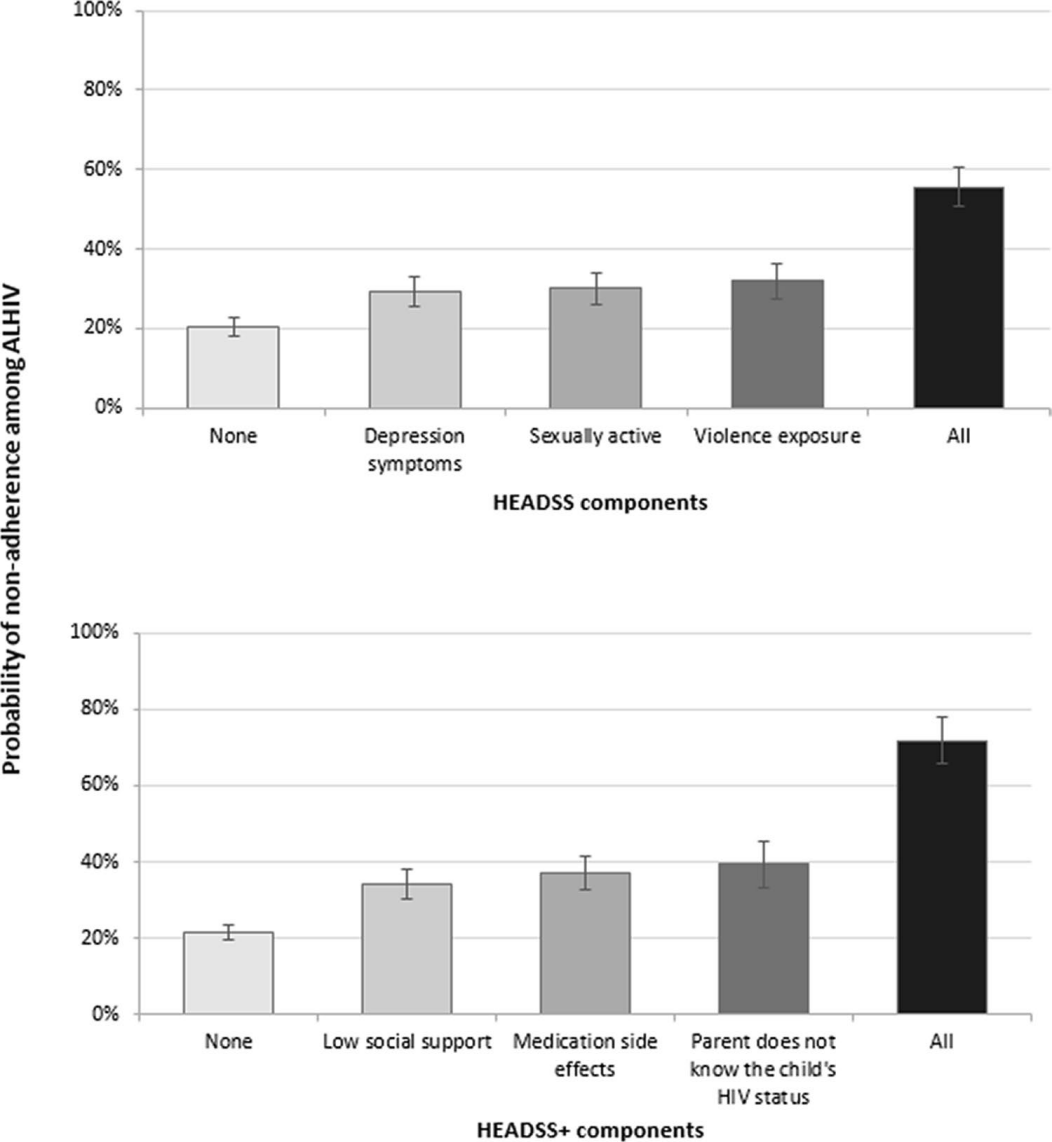
Adjusted predicted probabilities of non-adherence for HEADSS and HEADSS+

To illustrate the magnitudes, we report predicted probabilities using the coefficients from non-penalised regression and assuming that the distribution of all the factors remained the same among adolescents (see Fig. 7). For the HEADSS constructs, if adolescents report no violence exposure, no depression symptoms, and no sexual activity we would expect about 20.4% to be non-adherent to ART. Conversely, if an adolescent was experiencing violence, depression, and was sexually active, we would expect 55.6% to be non-adherent to ART.

For the HEADSS+ constructs, if adolescents report no medication side-effects, high social support and their parents know about their HIV-status, we would expect about 21.6% to be non-adherent to ART. Conversely, if an adolescent was experiencing medication side-effects, low social support and their parents do not know about their HIV, we would expect 71.8% to be non-adherent to ART.

### Sensitivity Analyses

The results of the sensitivity check were in congruence with our variable selection on the full dataset. In brief, the models that do not model clustering of time-specific observations within individuals and do not model the effect of time period provide the same top three variables within each model.

## Discussion

This study identifies simple constructs for identifying risk of adolescent non-adherence within two widely-used checklists in Sub-Saharan Africa. These findings may help healthcare workers identify adolescents in greatest need of support, and pinpoint areas to consider integrating into adolescent HIV care. Findings showed the three most-associated constructs from HEADSS: violence exposure, depression and sexual debut, were associated with increased ART non-adherence from 20.4 to 55.6%. The three most-associated constructs from HEADSS+: medication side-effects, low social support and parents unaware of adolescent HIV status, were associated with increased ART non-adherence from 21.6 to 71.8%. These findings indicate valuable indicators for ART non-adherence. HEADSS and HEADSS+ as established tools have additional value for HIV care, and may support adherence screening in high-burden settings.

Consultations with WHO’s Adolescent Service Delivery Working Group on HIV identified a need to plan operationalisation of these findings into practice. For example, adding an asterisk next to these three constructs could alert users about risks of non-adherence whilst administering the HEADSS and HEADSS+ checklists in routine clinical care. This could be flexible for contexts where both checklists are used together, or separately—for example, HEADSS+ items assume adolescents’ knowledge that they are HIV-positive, and so HEADSS may be more feasible for adolescents who are not aware of their own HIV-status.

The HEADSS and HEADSS+ tools could also be modified to expand their use in routine care. In clinical settings, posters on clinic walls might encourage adolescents to identify their own support needs, and peer supporters may be trained to administer these tools to identify at-risk adolescents. Whilst asking questions about medication side-effects and social support may be acceptable, more sensitive topics such as sexual activity or violence victimisation require closer consideration and timely referrals to further care, where needed. Some constructs within HEADSS and HEADSS+ may be easier to ask without the adolescent’s caregiver present. Evidence suggests that there may be important periods to use these checklists—for example as adolescents transition through stages of HIV services, and experience major life events such as parenthood or bereavement.

Our findings—that adolescent non-adherence is associated with side-effects, exposure to violence, mental health distress, sexual health and parent–child relationships—also have wider implications for HIV care services. Adherence counselling remains a primary response to anticipated or actual non-adherence, but in some studies shows lower effectiveness for adolescents than for adults [32]. This study supports increasing evidence for community-based and peer-support programs to improve adolescent adherence [33, 34]. Side-effect management is critical—especially in contexts with very limited treatment options. Integrating services for mental health, sexual and reproductive health, poverty reduction and parenting support into HIV care may be particularly valuable, as also found in recent studies from South Africa [35–37], Uganda [38, 39] and Botswana [7].

These considerations can be incorporated into provider training, support group curricula and community services, and use a preventative approach given high overall rates of mental health distress and violence exposure amongst adolescents living with HIV [40, 41]. Support for disclosure within families may benefit long-term adherence, and could be incorporated into future revisions of adherence counselling packages. There may also be opportunities for increasing digital delivery of evidence-based parenting programs and SRH services [42].

This study has several limitations. First, the study took place in one country, although comprising a very large sample, and including adolescents living in six provinces by the follow-up stage. Ideally, replication studies would test whether these constructs work similarly across the Sub-Saharan African region. Second, in the context of limited healthcare infrastructure, the viral load measures recorded in clinic files did not account for adolescents experiencing drug resistance despite good adherence: there was almost no routine testing or recording of viral resistance. Third, age and stage matter: we identified very high variability in adolescent adherence over time for each individual [35], perhaps reflecting the rapid developmental, social and sexual changes that characterise adolescence. This had implications for analysis, measurement and response. In the models, we focused on the concurrent relationships of the variables and used all time periods in the model together. However, key constructs identified in our analyses need to be tested in new samples as a predictive model for current and future adherence. There may be value in asking these brief screening questions regularly since we cannot expect consistency over time in adolescents’ experiences or adherence. From a service provision perspective, as adolescents’ circumstances and development undergo rapid changes, we need to ensure that mental health, sexual health and family support services are consistently available.

Fourth, the brief sets of constructs identified do not fully predict non-adherence, and we need to recognise heterogeneity amongst adolescents, especially when fitting models to explain a complex behaviour such as adherence. Our AUC was similar to that in another study of factors associated with adolescent adherence that used a Lasso approach [43]. Fifth, we note that there may be differences between how our study and different healthcare settings ask adolescents about constructs. For example, HEADSS does not specify how providers measure depression symptoms: we used a standardised brief child depression scale, but across countries and facilities there are likely to be differences in questions or scales used.

The study also has strengths. We were able to test an extensive set of constructs, mapped on two widely-used checklists, using validated and previously piloted tools for the region. Our sample included adolescents who were engaged and not engaged in HIV care, in 180 communities and over 70 government healthcare facilities, in an area with limited health and social services. Therefore, we were able to test associations of adherence within a population that reflects a wide range of adolescents receiving government-provided HIV care. Furthermore, our approach to selecting key constructs associated with non-adherence avoids multiple testing and relies on statistical significance, increasing reliability of results. Lastly, we had very high rates of adolescent retention in the study. Future research could explore whether similar constructs are associated with non-adherence in particular sub-groups of adolescents living with HIV, such as adolescent parents, adolescent key populations and adolescents with disabilities.

## Conclusions

These findings suggest critical constructs within two established checklists that can support identification of adolescents at high risk of non-adherence to ART. These constructs also highlight the close interlinkages between adolescents’ medical, social, familial and sexual wellbeing and their capacity to maintain adherence to ART and subsequent viral load. As we move towards approaches of differentiated care and precision programming, there may be a substantial benefit to integrating side-effect management, violence prevention, mental health, sexual health and family support into our screening and services for adolescents living with HIV.

### Supplementary Information

Below is the link to the electronic supplementary material.

Supplementary file1 (DOCX 20 KB)

## Data Availability

Please contact the authors for sharing data.

## Code Availability

We have made all R code available open-source online: https://github.com/marses/HEADSS.
