# Supplementary material for: Identifying Adolescents at Highest Risk of ART Non-adherence, Using the World Health Organization-Endorsed HEADSS and HEADSS+ Checklists
Source: AIDS Behav. 2023 Aug 17;28(1):141–53. doi: 10.1007/s10461-023-04137-6 (PMC10803572; doi:10.1007/s10461-023-04137-6)
Supplement: Supplementary file 1 — Supplementary file1 (DOCX 20 KB) [file 10461_2023_4137_MOESM1_ESM.docx]

# Supplementary materials

## Lasso Regression

In brief, Lasso regression puts a penalty on the regression coefficients (L1 penalty), shrinking the magnitude of some regression coefficients, while some are set to zero. This maximizes the log-likelihood $l\left( \beta\right)$ of the model while constraining the L1-norm of the parameter vector $\beta$ (regression coefficient). The Lasso estimate $\hat{\beta}$ is obtained by solving the following optimisation problem:

$\hat{\beta}=\underset{\beta}{\mathrm{argmax}} \left[ l\left( \beta\right)-{\lambda\left| \left| \beta\right| \right|}_{1} \right]$, (1)

where λ (lambda) is the regularisation parameter which determines the strength of L1-penalty [31].

When tuning parameter λ → 0 the resulting Lasso problem is reduced to the standard regression; as λ → ∞, the penalty becomes extremely large and regression estimates approach zero or become equal to zero. The λ is chosen in such a fashion as to avoid overfitting while avoiding excessive bias.Our analysis is primarily conducted in R using the following packages: *glmmLasso* for generalized linear mixed Lasso models, *glmnet*for standard Lasso and elastic net. We use the *margins* command in Stata v.16 to compute the predicted probabilities.

**Table 1 Supp: Multivariable random effects regression testing associations between self-reported ART adherence and undetectable viral load (number of participants N=842, observations N=1973).**

|  | **Non-detectable Viral Load (≤50 copies/ml)** | |
| --- | --- | --- |
| **Factors** | **aOR (95% CI)** | **p-value** |
| Self-reported adherence | 1.51 (1.11-2.05) | 0.008 |
| Age | 0.89 (0.83-0.96) | 0.003 |
| Female | 1.25 (0.86-1.82) | 0.237 |
| Rural residence | 0.67 (0.45-0.99) | 0.042 |
| Double orphan | 1.14 (0.74-1.74) | 0.549 |
| Informal housing | 0.94 (0.6-1.46) | 0.767 |
| Horizontal HIV acquisition | 0.77 (0.45-1.32) | 0.341 |
| aOR- adjusted odds ratio; 95% CI-95% confidence interval; This regression also controls forthe time of the survey. n1=746, n2=656 and n2=578 had a viral load record at each time of the survey (Wave 1, 2 and 3) respectively. | | |

## Questionnaire items used to measure the selected constructs/variables

### HEADSS aligned items

**No emotional or physical violence experience**

Adolescent experienced any of the following:

1. Someone used a stick, belt, other hard item to hit you
2. Someone slapped, punched, hit, etc. so that you were hurt/had marks
3. Someone in home/family has threatened to hurt you
4. Someone in home/family has said they would call ghosts or evil spirits
5. Someone in home/family has told you they wished they did not have to look after you
6. Someone in home/family has made you feel unwelcome in the home
7. Someone in family has said that you would be sent away out of the house
8. Someone in home/family has called you dumb, lazy, or other names
9. Someone in family has insulted members of your family that have passed away
10. Someone in home/family has threatened to leave you and n come back

**No depression symptoms (measured as any symptom endorsed from the depression scale (CDI-10):**

1. Frequency of sadness: How have you felt in the last 2 weeks?

- I am sad once in a while
- I am sad many times
- I am sad all the time

1. Feelings about appearance: How have you felt in the last 2 weeks?

- I look OK
- There are some bad things about my looks
- I look ugly

1. Feelings toward self: How have you felt in the last 2 weeks?

- I like myself
- I do not like myself
- I hate myself

1. Frequency of loneliness: How have you felt in the last 2 weeks?

- I do not feel alone
- I feel alone often
- I feel alone all the time

1. Self-evaluation: How have you felt in the last 2 weeks?

- I do most things OK
- I do many things wrong
- I do everything wrong

1. Friends: How have you felt in the last 2 weeks?

- I have enough friends
- I have some friends but wish I had more
- I don’t have any friends

1. Frequency of crying: How have you felt in the last 2 weeks?

- I feel like crying once in a while
- I feel like crying many days
- I feel like crying y day

1. Feelings of love: How have you felt in the last 2 weeks

- I am sure that somebody loves me
- I am not sure if anybody loves me
- Nobody really loves me

1. Bothered frequency: How have you felt in the last 2 weeks?

- Things bother me once in a while
- Things bother me many times
- Things bother me all the time

1. Personal outlook: How have you felt in the last 2 weeks?

- Things will work out for me OK
- I am not sure if things will work out for me
- Nothing will work out for me

**Sexual debut (**Participant has had sex). Based on whether the participant reported an age for vaginal and/or anal intercourse, OR had oral intercourse, OR admitted to currently using condoms, OR is currently using other forms of contraception, OR has been pregnant)

1. How old were you when you first had sex?
2. How old were you when someone first used their mouth to kiss your private parts (vagina)?
3. How old were you when you had sex in your bum?
4. Are you or your partner using anything to prevent getting pregnant?
5. How many times have you been pregnant?

### HEADSS+ aligned items

**No side effects from ARTs/medication**

- 1. In the last year, how often have you felt that taking ARVs or HIV medicine/taking pills caused you to have other physical symptoms (e.g., rash, headache, getting fat in unusual places, nausea, vomiting, diarrhoea)? (0-never, 1-sometimes, 2-often)

**High social support**

Medical Outcomes Study Social Support Survey (MOSS-SS)

People sometimes look to others for friendship and support.

1. How often do you have: Someone you can count on to listen when you need to talk."
2. How often do you have: Someone to give you good advice about a crisis."
3. How often do you have: Someone to share your most private worries and fears with."
4. How often do you have: Someone to turn to for suggestions about how to deal with personal problems."
5. How often do you have: Someone to help you if you were confined to bed."
6. How often do you have: Someone to take you to the doctor if you needed it."
7. How often do you have: Someone to prepare your meals if you were not well.

(0-never, 1-sometimes, 2-always; overall score classified as high (always have social support in all areas) versus low)

**Parents know about HIV/ARTs**

How much information about your health you share with Parent or Caregiver?

1. I don’t have a parent or caregiver
2. They don’t know anything about my health, illness or medication
3. They suspect something/ know from other sources
4. They know I am sick but they don’t know what I have
5. They know I am taking medication, but not what medication
6. They know about my HIV status
7. I talk to them about my HIV status
8. They know that I am taking ARVs/ medication, and the type of medication

9. I talk to them about my struggles with taking ARVs/medicine

(selecting multiple answers was possible; answers were grouped as selecting adolescents who selected responses 6, 7, 8 or 9 versus all other responses)
